# Biallelic loss-of-function variants in *KCNJ16* presenting with hypokalemic metabolic acidosis

**DOI:** 10.1038/s41431-021-00883-0

**Published:** 2021-04-12

**Authors:** Bryn D. Webb, Hilary Hotchkiss, Pankaj Prasun, Bruce D. Gelb, Lisa Satlin

**Affiliations:** 1 Department of Genetics and Genomic Sciences, Icahn School of Medicine at Mount Sinai, New York, NY USA; 2 Department of Pediatrics, Icahn School of Medicine at Mount Sinai, New York, NY USA; 3 Mindich Child Health and Development Institute, Icahn School of Medicine at Mount Sinai, New York, NY USA

**Keywords:** Paediatric kidney disease, Genetics research

## Abstract

*KCNJ16* encodes K_ir_5.1 and acts in combination with K_ir_4.1, encoded by *KCNJ10*, to form an inwardly rectifying K^+^ channel expressed at the basolateral membrane of epithelial cells in the distal nephron. This K_ir_4.1/K_ir_5.1 channel is critical for controlling basolateral membrane potential and K^+^ recycling, the latter coupled to Na-K-ATPase activity, which determines renal Na^+^ handling. Previous work has shown that *Kcnj16*^−/−^ mice and SS^*Kcnj16*−/−^ rats demonstrate hypokalemic, hyperchloremic metabolic acidosis. Here, we present the first report of a patient identified to have biallelic loss-of-function variants in *KCNJ16* by whole exome sequencing who presented with chronic metabolic acidosis with exacerbations triggered by minor infections.

## Introduction

Basolateral K^+^ channels in the aldosterone-sensitive distal nephron (ASDN), including the distal convoluted tubule (DCT) and the cortical collecting duct, are essential for controlling basolateral membrane potential and in recycling of K^+^ taken up into the cell by the basolateral Na-K-ATPase, the ubiquitous pump that drives transepithelial Na^+^ absorption [1]. *KCNJ16* encodes K_ir_5.1, which itself does not conduct K^+^, but which forms a heterotetramer with K_ir_4.1, encoded by *KCNJ10* [2]. The K_ir_4.1/K_ir_5.1 channel, the predominant basolateral K^+^ channel in ASDN, is an inwardly rectifying potassium channel that is exquisitely sensitive to intracellular pH within the physiological range.

The critical role of renal *KCNJ16* in maintaining electrolyte homeostasis and blood pressure has been revealed by studying both mouse and rat animal models with deletion of this gene. In the absence of functional K_ir_5.1, K_ir_4.1 forms a homomeric channel with enhanced K^+^ conductance and significantly diminished pH sensitivity. *Kcnj16*^−/^^−^ mice survive to adulthood but have an ~15% reduced body weight compared to wild-type controls. *Kcnj16*^−/−^ mice are polyuric and display a hypokalemic, hyperchloremic metabolic acidosis with increased urinary excretion of K^+^, calcium, and magnesium. Glomerular filtration rates are normal. The hypokalemia is associated with exaggerated renal Na^+^ absorption in the DCT. Systolic blood pressure and 24-h urine aldosterone excretion were similar between *Kcnj16*^−/−^ and wild-type mice [3]. A *Kcnj16*^−/−^ rat model was also created using Dahl SS rats, which develop severe hypertension when fed a high-salt diet. SS^*Kcnj16*−/−^ rats exhibit salt wasting, hypomagnesemia, and hypokalemia, and in contrast to normotensive *Kcnj16*^−/−^ mice, do not exhibit an upregulation of K_ir_4.1 homomeric channels on the basolateral membrane [4]. These rats also exhibit hyperventilation at rest in response to chronic hyperchloremic metabolic acidosis [5]. Aldosterone and angiotensin peptides are increased in SS^*Kcnj16*−/^^−^ rats compared to SS^WT^ rats, and these rats have an altered renin–angiotensin–aldosterone system response to high-salt and high-K^+^ diets [2].

Biallelic loss-of-function variants in *KCNJ10* (K_ir_4.1) have previously been reported to cause SESAME syndrome, which presents with onset of seizures in infancy, ataxia, sensorineural hearing loss, persistent hypokalemia, metabolic alkalosis, and hypomagnesemia. Plasma renin and aldosterone are increased in the absence of hypertension [6, 7]. Here, we report the association of biallelic loss-of-function variants in *KCNJ16* with a hypokalemic metabolic acidosis phenotype in a 2-year-old female patient, similar to that seen in rodent loss-of-function models.

## Materials and methods

In an effort to identify a possible causative genetic etiology for the proband, the family was enrolled into our Mount Sinai IRB-approved research study. Written informed consent was obtained, and all investigations were conducted in accordance with the principles of the Declaration of Helsinki.

### Whole exome sequencing

Genomic DNA was obtained from peripheral blood. Whole exome sequencing was performed on the proband and both parents at Genewiz (South Plainfield, NJ, USA). An Agilent SureSelect Exome kit (v6) was used for library preparation, and sequencing was performed on an Illumina HiSeq 2500 instrument (Illumina, San Diego, CA, USA) with 100-bp, paired-end reads. Alignment and variant calling was completed with an in-house pipeline that utilizes bwa-mem and GATK. 88.66% of the target had ≥30× coverage for the proband’s sample, and 88.25% and 88.89% for the mother and father’s samples, respectively. Variants were filtered with Ingenuity Variant Analysis (Qiagen, Redwood City) based on confidence, frequency, predicted deleteriousness (coding or splicing change, CADD score ≥20, and variants listed in HGMD were included), and genetic analysis (de novo or recessive inheritance considered). This filtering strategy resulted in the identification of 33 variants in 31 genes (Supplementary Table 1). All of these variants were considered, and knowledge of gene function was reviewed. The identified *KCNJ16* variant was confirmed by Sanger sequencing in both proband and parental samples. The *KCNJ16* variant was submitted to LOVD database (https://databases.lovd.nl/shared) (submission ID: 0000327632, Individual ID: 00326418).

## Results

### Clinical case description

The proband was a grossly nondysmorphic 2-year-old female, height at the 53rd percentile and weight at the 56th percentile, daughter of a consanguineous union who was born at full term by normal standard vaginal delivery and presented with dehydration and acidosis precipitated by minor illnesses. At 21 months of age, the patient presented to the emergency room with a 1-day history of fever, rhinorrhea, foul-smelling urine, and decreased oral intake and was found to be Influenza B positive. Of note, the parents denied history of diarrhea. Initial vital signs in the emergency department were normal for age and included heart rate of 140 beats/min, respiratory rate of 26 breaths/min, and blood pressure of 86/58 mmHg. Physical exam was notable only for rhinorrhea, and no increased work of breathing was noted. Initial labs were significant for an extremely low serum tCO_2_ at 7 mmol/L. Serum sodium was 129 mmol/L (reference range: 136–145), potassium was 3.2 mmol/L (reference range: 3.5–5.1), chloride was 111 mmol/L (reference range: 98–108), blood urea nitrogen was 14 mg/dL (reference range: 6–23), serum creatinine was 0.32 mg/dL (reference range: 0.24–0.41), serum calcium level was 8.6 mg/dL (reference range: 8.6–10.3), and serum magnesium level was 1.9 mg/dL (reference range: 1.6–2.3). Intravenous hydration was initiated. In addition, urinalysis revealed a pH of 6.0, specific gravity of 1.018 mOsm/kg, ketones at 80 mg/dL, and 30 mg/dL of protein. Urine was negative for blood, leukocyte esterase, and nitrites. A complete blood count revealed a normal white blood count at 12.82 × 10^3^/mcL and differential was notable for 15% bands. Chest X-ray was normal. The patient was hospitalized for continued intravenous hydration and noted to have a persistent metabolic acidosis despite aggressive hydration, improved appetite, and resolution of symptoms. During the course of the hospitalization, urine electrolytes were obtained, which included a urine calcium:creatinine ratio of 0.06 (normal < 0.2). Liver enzymes were noted to be elevated to a maximum of AST 583 u/L (reference range: 4–32) and ALT 390 u/L (reference range: 4–33). A single parathyroid hormone level during the hospitalization was noted to be low at 11.39 pg/ml (reference range: 15–65 pg/mL). In addition, the patient was noted to have altered mental status as demonstrated by an abnormal gait. Computerized tomography of the head without contrast revealed extensive bilateral symmetric calcification within the basal ganglia, subcortical white matter of both cerebral hemispheres, and within the cerebellar hemispheres. Magnetic resonance imaging of the brain also noted widespread intracranial calcifications. Mental status gradually returned to baseline over the course of several days, suggesting her transient alternation in mental status was due to the presenting infectious process and biochemical abnormalities. Abdominal and renal ultrasound were normal, and renal calcifications were not seen. The patient was discharged home on citric acid/sodium citrate 20 mEq by mouth three times daily (equivalent to 4 mEq/kg/day). At baseline, and while on citric acid/sodium citrate, the patient continued to demonstrate a persistent metabolic acidosis.

### Whole exome sequencing

**Fig. 1:**
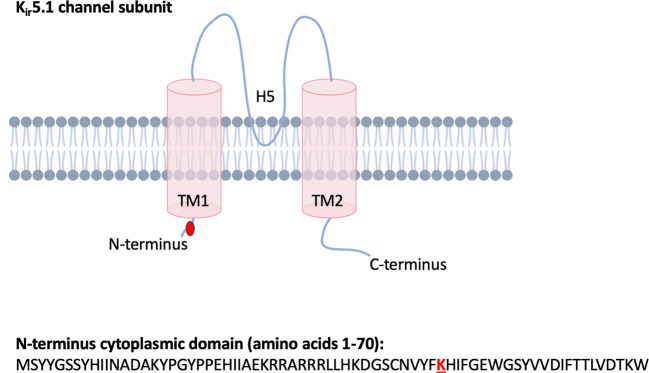
A cartoon of the KCNJ16 protein (K_ir_5.1) is shown. K48 is located in the N-terminus domain.

By whole exome sequencing, we identified a homozygous variant in *KCNJ16* c.142A>T; p.(Lys48*) (NM_001291624.1; rs142011800; GRCh37.p13 chr17 NC_000017.10:g.68128370A>T) in the proband, which was heterozygous in both parents consistent with autosomal recessive inheritance. This variant has a high CADD score of 36.0, and is in gnomAD at very low frequency (6/282858 alleles [all individuals of African ancestry]; no homozygotes reported). Lys48 is located in the cytoplasmic 5′ terminus region prior to the first transmembrane domain (Fig. 1); c.142A>T *KCNJ16* mRNA transcripts would most likely result in nonsense-mediated decay. Per GTEx Project data, KCNJ16 is expressed in the kidney, thyroid, and pancreas, and with low level expression in the brain. *KCNJ16* is not currently known to be an OMIM-disease causing gene.

## Discussion

Here, we present the first report of an individual with biallelic loss-of-function variants in *KCNJ16* presenting with the expected phenotype of hypokalemic metabolic acidosis. As has been reported for the *Kcnj16*^−/−^ mouse model and the SS^*Kcnj16*−/−^ rat models, our patient exhibits chronic hypokalemic metabolic acidosis, which is exacerbated by minor infections or illness. Serum chloride was mildly elevated at presentation. Like the *Kcnj16*^−^^/−^ mice, our patient has normal blood pressure.

A noteworthy finding in our patient is diffuse intracranial calcifications (ICCs), including in the basal ganglia, which have not been noted in either the *Kcnj16*^−/−^ mice or SS^*Kcnj16*−/−^ rat models, though they may not have been assessed for. Hypoparathyroidism is a common cause of basal ganglia calcification with more diffuse ICCs reported rarely [8, 9]. While a diagnosis of hypoparathyroidism is supported in our patient by the borderline low serum calcium concentrations and a low level of parathyroid hormone, the patient did not have elevated serum phosphate levels, which would be expected in the face of a low parathyroid hormone level. It is the hyperphosphatemia that contributes to mineral deposition and calcification in a number of extraskeletal sites, including the basal ganglia. Alternatively, the patient’s ICCs may represent an incidental finding reflecting an infectious process early in life or may reflect a co-occuring metabolic or genetic disease. With identification of additional patients with this disorder, we will be able to ascertain if ICC is a frequent component to this syndrome. The proband is being treated with citric acid/sodium citrate and with close monitoring when there are signs or symptoms of infection.

## Supplementary information

Supplemental Table 1.
